# Prognostic value of baseline genetic features and newly identified *TP53* mutations in advanced breast cancer

**DOI:** 10.1002/1878-0261.13297

**Published:** 2022-08-15

**Authors:** Lanxin Zhang, Siwen Sun, Xiaotian Zhao, Jingwen Liu, Yang Xu, Lingzhi Xu, Chen Song, Na Li, Jing Yu, Shanshan Zhao, Peiyao Yu, Fengqi Fang, Jiping Xie, Xuening Ji, Ruoying Yu, Qiuxiang Ou, Zuowei Zhao, Man Li

**Affiliations:** ^1^ Department of Oncology The Second Hospital of Dalian Medical University China; ^2^ Geneseeq Research Institute Nanjing Geneseeq Technology Inc. China; ^3^ Department of Oncology First Affiliated Hospital of Dalian Medical University China; ^4^ Department of Breast and Thyroid Surgery Affiliated Zhongshan Hospital of Dalian University China; ^5^ Department of Oncology Affiliated Zhongshan Hospital of Dalian University China; ^6^ Department of Breast Surgery The Second Hospital of Dalian Medical University China

**Keywords:** advanced breast cancer, baseline genetic features, prognosis, *TP53* mutations

## Abstract

Approximately 30% of breast cancer (BC) patients suffer from disease relapse after definitive treatment. Monitoring BC at baseline and disease progression using comprehensive genomic profiling would facilitate the prediction of prognosis. We retrospectively studied 101 BC patients ultimately experiencing relapse and/or metastases. The baseline and circulating tumor DNA‐monitoring cohorts included patients with baseline tumor tissue and serial plasma samples, respectively. Samples were analyzed with targeted next‐generation sequencing of 425 cancer‐relevant genes. Of 35 patients in the baseline cohort, patients with *TP53* mutations (*P* < 0.01), or *CTCF/GNAS* mutations (*P* < 0.01) displayed inferior disease‐free survival, and patients harboring *TP53* (*P* = 0.06) or *NOTCH1* (*P* = 0.06) mutations showed relatively poor overall survival (OS), compared to patients with wild‐type counterparts. Of the 59 patients with serial plasma samples, 11 patients who were newly detected with *TP53* mutations had worse OS than patients whose *TP53* mutational status remained negative (*P* < 0.01). These results indicate that an inferior prognosis of advanced breast cancer was potentially associated with baseline *TP53*, *CTCF,* and *NOTCH1* alterations. Newly identified *TP53* mutations after relapse and/or metastasis was another potential prognostic biomarker of poor prognosis.

## Graphical abstract

This study provided insights into the prognostic value of genetic features obtained at baseline and/or during dynamic monitoring advanced breast cancer (ABC). Baseline altered *TP53*, *CTCF*, *GNAS*, and *NOTCH1* genes were negative prognostic factors, and the evolutionary pattern of *TP53* mutation during ABC treatment could potentially serve as a prognostic factor for recurrent patients.

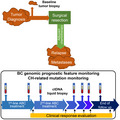

**Abbreviations**
ABC: advanced breast cancer
BC: breast cancer
cfDNA: cell‐free DNA
CI: confidence interval
ctDNA: circulating tumor DNA
DFS: disease‐free survival
FFPE: formalin‐fixed paraffin‐embedded
HER2: human epidermal growth factor receptor 2
HR: hormone receptor
NGS: next‐generation sequencing
OS: overall survival
PFS: progression‐free survival
TNBC: triple‐negative breast cancer
VAF: variant allele frequency

## 1 Introduction

Breast cancer (BC) is the most frequently diagnosed cancer and the leading cause of cancer‐related death in women [1]. Due to the advancement of early cancer detection technologies and various anti‐BC therapies, the 5‐year survival rate of BC is as high as 90% [1, 2]. Nevertheless, almost 30% of patients who reach complete response after primary treatments experienced relapse [3], and the survival rate in advanced breast cancer (ABC) patients is only approximately 30% [4].

Prognostic biomarkers have played an important role in BC treatment by informing the probability of recovery, helping patients avoid overtreatment and related adverse effects, and stratifying patients in clinical research [5, 6]. Research has been focusing on genomic DNA from tumor to identify prognostic genetic biomarkers, in addition to routine clinical and histological factors. For instance, the alteration of *TP53* gene, detected in over 20% of BC patients, has been identified as a strong predictor of poor BC survival [7, 8, 9]. *MYC* gene amplification [10, 11], related to BC development and progression, has also be considered as a powerful negative prognostic factor, especially in BC patients with node‐negative and hormone receptor (HR)‐negative disease. Somatic aberrations of other genes, such as *RB1* and *ERBB2* [12, 13, 14], can also facilitate the prediction of BC patients' prognosis. Similarly, germline mutations, such as *BRCA1* and *BRCA2* mutations [15, 16], were associated with poorer prognosis in BC patients [17, 18, 19, 20]. However, tissue biopsy can hardly capture the entire genomic profile of tumor due to the high heterogeneity of BC. It is also difficult to get each metastatic tumor tissue sample among ABC patients due to biopsy compliance and the cost of time and money. Alternatively, liquid biopsy can overcome such limitations and track the clonal evolution of patients undergoing various treatments [21, 22].

Circulating tumor DNA (ctDNA) mainly released from apoptotic and/or necrotic tumor cells [23] serves as an emerging biomarker for BC screening, diagnosis, and prognosis [24, 25, 26]. Owing to the accessibility of ctDNA and the high sensitivity of next‐generation sequencing (NGS) technology, ctDNA is used to dynamically monitor the disease progression of early BC and the drug resistance in ABC [27, 28]. However, the association between the genetic evolution of ABC and patients' prognosis has not been comprehensively investigated.

In this study, we aimed to identify prognostic genetic features among 101 BC patients who ultimately developed relapse and/or metastases. We tracked their clonal evolution by conducting ctDNA monitoring and analysis using a broad NGS panel targeting 425 cancer‐related genes. Our results provided insights into the prognostic value of various genetic features obtained at baseline and/or during dynamic monitoring ABC.

## 2 Materials and methods

### 2.1 Patients

**Fig. 1:**
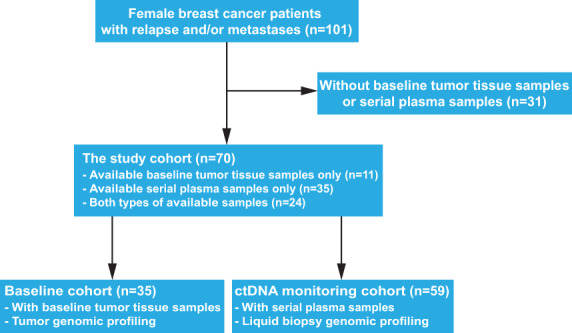
The flowchart of enrollment and study cohorts. A total of 70 participants were enrolled in the baseline cohort and/or the ctDNA monitor cohort. Thirty‐five patients with available baseline tumor tissue samples were included in the baseline cohort, and 59 patients with serial plasma samples during ABC treatment were included in the ctDNA monitoring cohort. Twenty‐four patients were included in both the baseline and ctDNA monitoring cohort.

We retrospectively studied a total of 101 Chinese female BC patients who were diagnosed and treated in the Second Hospital of Dalian Medical University (*n* = 77), the First Hospital of Dalian Medical University (*n* = 14), and the Affiliated Zhongshan Hospital of Dalian University (*n* = 10), between 1995 and 2020. All 101 enrolled BC patients developed distant metastases at initial diagnosis, or relapse and/or metastases after the primary treatment. Of these 101 ABC patients, 31 had neither available baseline formalin‐fixed paraffin‐embedded (FFPE) tumor tissue samples nor serial plasma samples after relapse and/or metastases and were thus excluded from further analyses. Of the remaining 70 patients, those with baseline FFPE tumor tissue samples were grouped as the ‘baseline cohort’, and those with serial plasma samples after relapse and/or metastases were referred as the ‘ctDNA monitoring cohort’ (Fig. 1). Patient's HR and human epidermal growth factor receptor 2 (HER2) status were determined by immunohistochemistry or fluorescence *in situ* hybridization [29]. This study was conducted in compliance with the Declaration of Helsinki and approved by the ethics committees of the three hospitals involved (Approval ID:2021v110). Informed written consent was provided by each participant.

### 2.2 Sample collection and targeted NGS

Formalin‐fixed paraffin‐embedded baseline tumor tissue biopsies of the primary tumor were collected. Genomic DNA from FFPE samples was extracted using QIAamp DNA FFPR Tissue Kit (QIAGEN, Hilden, Nordrhein Westfalen, Gernmany). Plasma and leukocyte (normal blood controls) were separated from peripheral blood collected in EDTA‐coated tubes (BD, Franklin Lakes, NJ, USA) by centrifuging at 1800 ***g*** for 10 min, within 2 h of blood sample collection. Cell‐free DNA (cfDNA) from plasma was extracted using QIAamp Circulating Nucleic Acid Kit (QIAGEN, Hilden, Nordrhein Westfalen, Gernmany).

All samples were analyzed with NGS tests at a centralized clinical testing center (Nanjing Geneseeq Technology Inc., Nanjing, Jiangsu, China), according to the protocols approved by the ethics committees of three hospitals. Sequencing libraries were prepared using the KAPA Hyper Prep Kit (KAPA Biosystems, Hertfordshire, UK). In brief, fragment genomic DNA or cfDNA underwent end‐repairing, A‐tailing, adapter ligation, size selection using Agencourt AMPure XP beads (Beckman Coulter, Brea, CA, USA), and polymerase chain reaction amplification sequentially, followed by purification. Target enrichment was performed using customized xGen lockdown probes panel (Integrated DNA Technologies, Coralville, IA, USA) targeting 425 cancer‐relevant genes, Human cot‐1 DNA (Life Technologies, Carlsbad, CA, USA) and xGen Universal Blocking Oligos (Integrated DNA Technologies, Coralville, IA, USA). Enriched libraries were sequenced on Illumina Hiseq4000 NGS platforms (Illumina, San Diego, CA, USA). The average coverage depths were at least 500× for tumor tissue genomic DNA, 3000× for cfDNA, and 100× for normal blood controls.

### 2.3 Mutation calling and data processing

FASTQ file quality control was conducted by trimmomatic, removing leading/trailing low quality (reading < 15) or N bases [30]. Sequencing data were then aligned to the reference human genome (build hg19) using the Burrows–Wheeler Aligner (bwa‐mem) [31] and processed using the Picard suite and the Genome Analysis Toolkit (gatk) [32]. Somatic mutations were called using VarScan2 and HaplotypeCaller/UnifiedGenotyper in gatk [33]. A somatic mutation call was retained when it had at least 1% (for tumor tissue samples) or 0.5% (for plasma samples) variant allele frequency (VAF) and at least three unique reads on different strands with good quality scores, followed by manual inspection in Integrative Genomics Viewer Software (igv, Broad Institute). Gene fusions and copy number variations were analyzed using factera and adtex, respectively [34, 35]. The cut‐offs of retaining copy number variation were 1.6 for amplifications and 0.6 for deletions.

### 2.4 Statistical analysis

Disease‐free survival (DFS) of patients who were in stage I–III at initial diagnosis and successfully received surgical resection was defined as the time from the surgical resection to the relapse and/or metastases. Overall survival (OS) was defined as the time from the initial diagnosis of primary BC to death or the end of follow‐up. Kaplan–Meier curves for OS were generated, and log‐rank tests were used to compare differences between independent subgroups. Cox proportional hazards models were used to estimate hazard ratios of prognostic factors, and the proportionality of hazards was assessed using log(−log) survival plots. Accounting for the left‐truncation issue and the immortal time bias was implemented [36], and the correction was applied to the delayed entry between diagnosis and performing genomic sequencing. The association between baseline genetic alterations and progression‐free survival (PFS)/OS was analyzed for altered genes identified in at least two patients in the baseline cohort, and FDR correction for the multiple comparison issue was applied over these genes as well as baseline clinical characteristics. Genetic alterations showing significant association with prognosis in univariate analyses, as well as clinical characteristics with potential confounding effects were included in multivariable analyses, and Cox regression models with Firth's penalized likelihood were used owing to the sparsity of the dataset [37]. Chi‐squared test or Fisher's exact test was performed to compare the frequencies between independent subgroups. All quoted *P*‐values were two‐tailed, with values < 0.05 considered to be statistically significant. Data were analyzed using r language (version 4.0.3, R Foundation for Statistical Computing, Vienna, Austria) with *survival* and *coxphf* packages.

## 3 Results

### 3.1 Patient overview

The study cohort of 70 Chinese ABC patients included 11 with baseline tumor tissue samples only, 35 with serial plasma samples only, as well as 24 patients with both baseline tissue and serial plasma samples, and their clinical characteristics were summarized in Table 1. The median age at diagnosis of the study cohort was 48 (range, 28–74) and 33 (47.14%) patients were 50 years or older. Of these 70 patients, 38 (54.29%) were classified as the HR‐positive/HER2‐negative subtype at initial diagnosis. Most patients (64/70, 91.43%) were in stage I–III at initial diagnosis, and they received surgical resection as the primary therapy. The most frequent metastatic sites included bone (43/70, 61.43%), liver (42/70, 60.00%), and lymph nodes (41/70, 58.57%). After relapse and/or metastases, 46 (65.71%) patients experienced at least 4‐line ABC treatment. The baseline cohort included 35 patients with available baseline tumor tissue biopsies, and the ctDNA monitoring cohort included 59 patients with serial plasma samples after relapse and/or metastases. No significant differences in clinical characteristics were detected between these two cohorts, even though fewer triple‐negative breast cancer (TNBC) patients (16.95% vs. 28.57%) and more patients with at least four lines of ABC treatment (72.88% vs. 51.43%) were observed in the ctDNA monitoring cohort (Table 1).

**Table 1:** Overview of patient demographics and clinical characteristics. The baseline cohort included patients with available baseline tumor tissue samples; the ctDNA‐monitoring cohort included patients with serial plasma samples after relapse and/or metastases.

Characteristics	Study cohort (*n* = 70)	Baseline cohort (*n* = 35)	ctDNA monitoring cohort (*n* = 59)	*P*‐value^a^
Age at initial diagnosis, median (range), year	48 (28–74)	51 (30–68)	48 (28–74)	
Age at initial diagnosis, no. (%)				
< 50 years	37 (52.86)	16 (45.71)	32 (54.24)	0.52
≥ 50 years	33 (47.14)	19 (54.29)	27 (45.76)	
Subtype at initial diagnosis, no. (%)				
Luminal (HR+/HER2−)	38 (54.29)	18 (51.43)	31 (52.54)	0.56
Luminal (HR+/HER2+)	6 (8.57)	2 (5.71)	6 (10.17)	
HER2‐enriched (HR−/HER2+)	12 (17.14)	5 (14.29)	12 (20.34)	
TNBC	14 (20.00)	10 (28.57)	10 (16.95)	
T stage at initial diagnosis, no. (%)				
T1	21 (30.00)	11 (31.43)	18 (30.51)	0.53
T2	29 (41.43)	14 (40.00)	26 (44.07)	
T3	10 (14.29)	7 (20.00)	6 (10.17)	
T4	9 (12.86)	2 (5.71)	8 (13.56)	
Unknown	1 (1.43)	1 (2.86)	1 (1.69)	
N stage at initial diagnosis, no. (%)				
N0	14 (20.00)	5 (14.29)	12 (20.34)	0.57
N1	19 (27.14)	8 (22.86)	17 (28.81)	
N2	13 (18.57)	10 (28.57)	9 (15.25)	
N3	23 (32.86)	11 (31.43)	20 (33.90)	
Unknown	1 (1.43)	1 (2.86)	1 (1.69)	
M stage at initial diagnosis, no. (%)				
M0	65 (92.86)	33 (94.29)	55 (93.22)	1.00
M1	5 (7.14)	2 (5.71)	4 (6.78)	
Clinical stage at initial diagnosis, no. (%)				
I	6 (8.57)	4 (11.43)	5 (8.47)	0.43
II	24 (34.29)	8 (22.86)	22 (37.29)	
III	34 (48.57)	21 (60.00)	27 (45.76)	
IV	6 (8.57)	2 (5.71)	5 (8.47)	
Primary therapy, no. (%)				
Surgical resection	64 (91.43)	31 (88.57)	53 (89.83)	0.88
Chemotherapy/radiotherapy	2 (2.86)	2 (5.71)	2 (3.39)	
Targeted	4 (5.71)	2 (5.71)	4 (6.78)	
Main metastatic location, no. (%)				
Bone	43 (61.43)	19 (54.29)	39 (66.10)	0.96^b^
Brian	13 (18.57)	6 (17.14)	10 (16.95)	
Liver	42 (60.00)	20 (57.14)	37 (62.71)	
Lymph node	41 (58.57)	15 (42.86)	34 (57.63)	
Pulmonary	31 (44.29)	16 (45.71)	25 (42.37)	
Soft tissue	26 (37.14)	13 (37.14)	21 (35.59)	
Lines of treatment for ABC, no. (%)				
1	10 (14.29)	7 (20.00)	5 (8.47)	0.14
2	7 (10.00)	6 (17.14)	5 (8.47)	
3	7 (10.00)	4 (11.43)	6 (10.17)	
≥ 4	46 (65.71)	18 (51.43)	43 (72.88)	
Lines of treatment for ABC, median (range)	5 (1–12)	4 (1–11)	5 (1–12)	

^a^ *P*‐value between the baseline cohort and the ctDNA‐monitoring cohort.

^b^ *P*‐value calculated by Pearson's Chi‐squared test.

### 3.2 Baseline prognostic genetic features

**Fig. 2:**
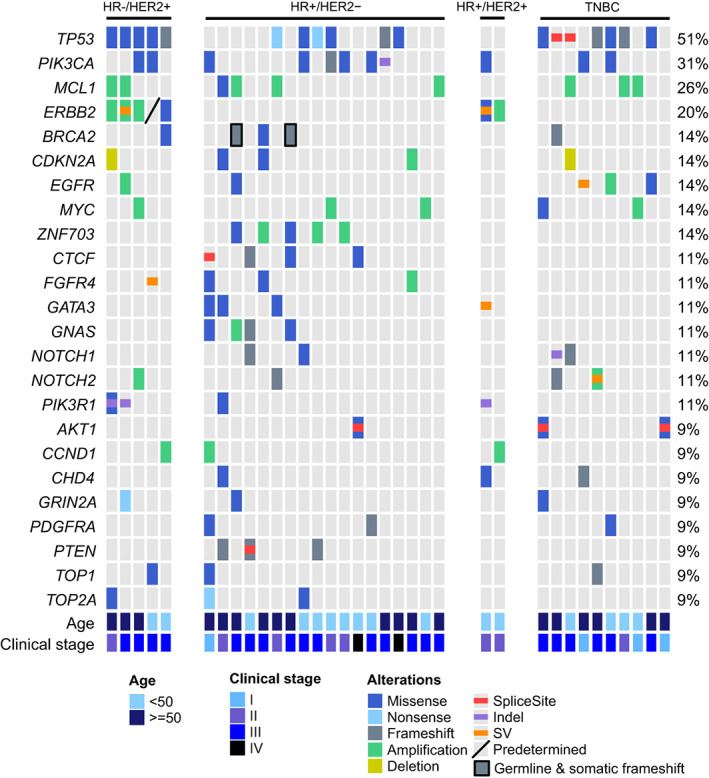
Genetic profile of the baseline cohort. Baseline tumor tissue samples of 35 baseline cohort patients were performed by tumor genomic profiling. Altered genes detected in at least 9% patients were displayed here, and the most frequently altered genes were *TP53*, *PIK3CA*, *MCL1*, and *ERBB2*. *TP53* alternations were enriched in HR‐negative patients, while *ZNF703*, *CTCF*, and *GNAS* alternations were only observed in HR‐positive/HER2‐negative patients.

We performed tumor genomic profiling on 35 baseline tumor tissue samples of patients in the baseline cohort. *TP53* alterations were most frequently observed (18/35, 51.43%), especially HR‐negative patients (*P* < 0.01, Table S1). Other top mutant genes included *PIK3CA*, *MCL1*, and *ERBB2*. Additionally, *ZNF703*, *CTCF*, and *GNAS* alterations were exclusively observed among the patients in HR‐positive/HER2‐negative subtype (Fig. 2), and the percentage of patients harboring *CTCF* alteration was 11% (4 of 35). Genetic alterations of 35 baseline tumor tissue samples were summarized in Table S2.

The relationship between baseline genetic features and patients' prognosis was investigated in the baseline cohort, and unadjusted hazard ratios were estimated by univariate Cox regression models. For DFS analyses, four patients who did not receive surgical resections were temporarily excluded. Of the remaining 31 patients receiving surgical resection as their primary therapy, patients with mutant *CTCF* gene were exactly the same individuals harboring *GNAS* mutations, while co‐mutation of *CTCF* and *GNAS* were not observed in the entire baseline cohort. Four frequently observed genetic aberrations were significantly associated with inferior DFS, including *TP53* mutations [hazard ratio = 3.14, 95% confidence interval (CI): 1.37–7.20, FDR‐corrected *P* = 0.03], *CTCF*/*GNAS* mutations (hazard ratio = 7.23, 95% CI: 1.78–29.38, FDR‐corrected *P* = 0.02), and *TOP1* mutations (hazard ratio = 7.21, 95% CI: 1.37–29.68, FDR‐corrected *P* = 0.02). Of note, patients with *NOTCH2* alterations appeared to have poorer DFS than those with wild‐type counterparts (hazard ratio = 3.44, 95% CI: 0.98–12.13, FDR‐corrected *P* = 0.20), while the *P*‐value and FDR‐corrected *P*‐value were larger than 0.05. Mutations in *TP53* gene (hazard ratio = 3.43, 95% CI: 0.90–13.11, FDR‐corrected *P* = 0.49) were potentially associated with inferior OS when compared to wild‐type counterparts. Patients carrying mutant *NOTCH1* gene also had relatively poor OS, compared to patients without *NOTCH1* mutations (hazard ratio = 3.52, 95% CI: 0.87–14.37, FDR‐corrected *P* = 0.49). The prognostic potential of clinical characteristics, including age, clinical stage, receptor status, neoadjuvant treatment, and postsurgery adjuvant chemotherapy were also investigated in the baseline cohort (Table 2).

**Table 2:** Unadjusted hazard ratios of baseline prognostic factors estimated by univariate cox regression models. Ref, reference.

Characteristics	No. of patients (%)	Hazard ratio (95% CI)	*P*‐value	*P*‐value with correction
Baseline prognostic factors of DFS				
*TP53* mutation	15 (48.39)	3.14 (1.37–7.20)	< 0.01^a^	0.03^a^
*CTCF/GNAS* mutation	3 (9.67)	7.23 (1.78–29.38)	< 0.01^a^	0.02^a^
*TOP1* mutation	3 (9.67)	7.21 (1.37–29.68)	< 0.01^a^	0.02^a^
*NOTCH2* alteration	3 (9.67)	3.44 (0.98–12.13)	0.05	0.20
Age at initial diagnosis	31 (100.00)	1.00 (0.96–1.05)	0.85	0.91
Clinical stage III vs. I/II	21 (60.00)	1.40 (0.66–2.95)	0.38	0.70
Subtype at initial diagnosis				
HER2‐enriched (HR−/HER2+)	4 (12.90)	Ref	Ref	Ref
HR+/HER2−	16 (51.61)	0.29 (0.09–0.93)	0.04^a^	0.20
HR+/HER2+	2 (6.45)	0.33 (0.06–1.91)	0.22	0.60
TNBC	9 (29.03)	0.61 (0.18–2.09)	0.43	0.73
Neoadjuvant therapy	8 (25.81)	1.68 (0.73–3.84)	0.22	0.60
Adjuvant chemotherapy	24 (77.42)	0.74 (0.31–1.76)	0.50	0.75
Baseline prognostic factors of OS				
*TP53* mutation	18 (51.43)	3.43 (0.90–13.11)	0.06	0.49
*NOTCH1* mutation	4 (11.43)	3.52 (0.87–14.37)	0.06	0.49
Age at initial diagnosis	35 (100.00)	1.00 (0.95–1.06)	0.97	0.97
Clinical stage III/IV vs. I/II	23 (65.71)	3.66 (1.03–12.97)	0.03^a^	0.49
Subtype at initial diagnosis				
HER2‐enriched (HR−/HER2+)	5 (14.29)	Ref	Ref	Ref
HR+/HER2−	18 (51.43)	0.31 (0.08–1.18)	0.09	0.53
HR+/HER2+	2 (5.71)	0.17 (0.01–1.92)	0.15	0.82
TNBC	10 (28.57)	0.38 (0.10–1.53)	0.17	0.82
Neoadjuvant therapy	10 (28.57)	1.39 (0.51–3.74)	0.52	0.91
Adjuvant chemotherapy	24 (68.57)	0.35 (0.13–0.97)	0.04^a^	0.49

^a^ Statistically significant.

**Fig. 3:**
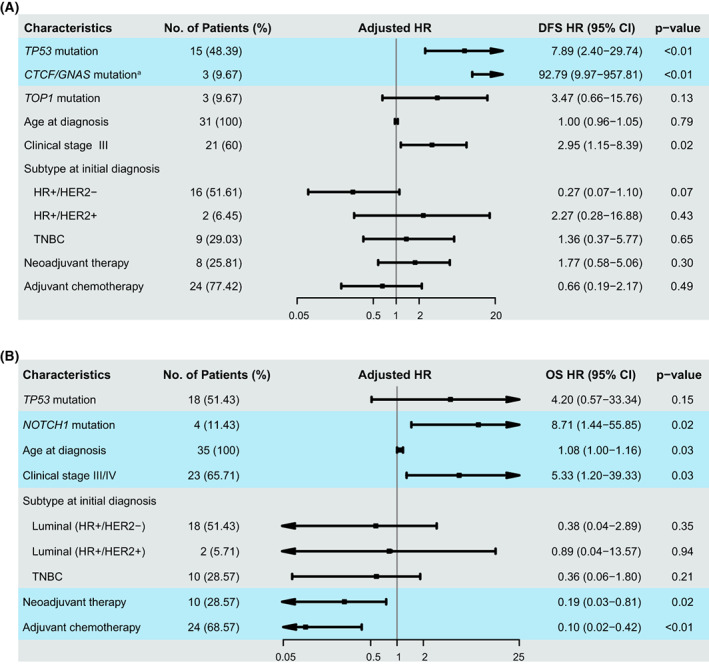
Baseline prognostic indicators and adjusted hazard ratios. Multivariable Cox regression models were fitted to control for confounding effects, and adjusted hazard ratios with 95% CIs and *P*‐values of all covariates in multivariable Cox regression models were shown. (A) Patients carrying *TP53* mutations, or *CTCF*/*GNAS* mutations had poor DFS. (B) Patients carrying *TP53* or *NOTCH1* mutations had relatively poor overall survival. ^a^Comutation was observed in the *CTCF* and *GNAS* gene in all three patients who were included in DFS analyses, so the effect of *CTCF* and *GNAS* mutations could not be separated.

For DFS, five clinical characteristics (age, clinical stage, receptor status, neoadjuvant, and postsurgery adjuvant therapy history) were included in the multivariable Cox regression model with Firth's penalized likelihood, to mitigate potential confounding effects. *TP53* mutations (adjusted hazard ratio = 7.89, 95% CI: 2.40–29.74) and *CTCF/GNAS* mutations (adjusted hazard ratio = 92.79, 95% CI: 9.97–957.81) were associated with inferior DFS (Fig. 3A). However, *TOP1* mutation was no longer significantly related with DFS (adjusted hazard ratio = 3.47, 95% CI: 0.66–15.76), even though its point estimate showed that patients with *TOP1* mutation were likely to have relatively poor prognosis after surgical resection. For OS, same potential confounders were adjusted for in the multivariable model. *NOTCH1* mutations (adjusted hazard ratio = 8.71, 95% CI: 1.44–55.85) were negatively related with OS in BC patients (Fig. 3B). Patient with *TP53* mutations appeared to have relatively poor OS than patients without (adjusted hazard = 4.20), while the 95% CI covered 1.

### 3.3 Serial monitoring of ctDNA mutational status

Next, we performed genomic profiling on serial plasma samples of 59 patients in the ctDNA monitoring cohort. Twenty‐four patients had both baseline tumor samples and serial ctDNA samples, and 19 of them had their first ctDNA tests within one month after relapse and/or metastases. ctDNA status were positive among 13 of 19 patients at the first ctDNA test, and the most frequently altered genes were *TP53* (8/19, 42.11%) and *PIK3CA* (5/19, 26.32%). Of note, during relapse and/or metastases, *TP53* mutations were newly identified in three patients whose baseline tumor tissue samples were negative for *TP53* mutations. Genetic alterations of serial plasma samples were summarized in Table S3.

**Fig. 4:**
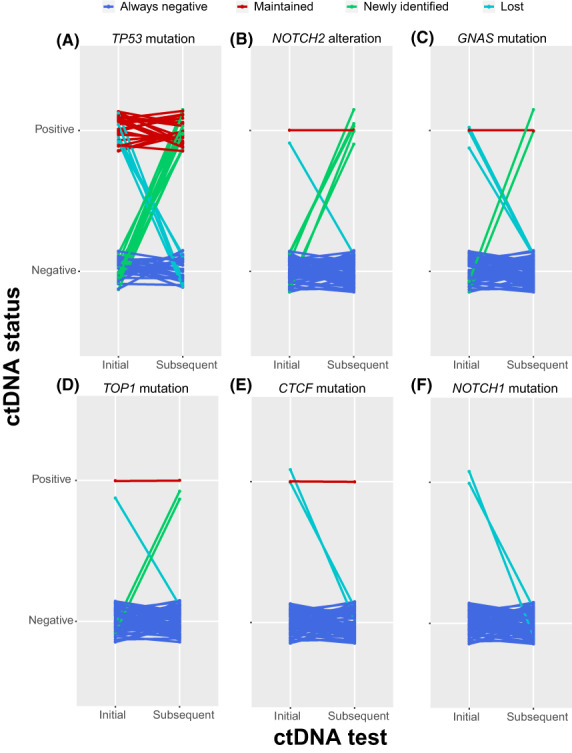
Evolutionary pattern of baseline prognostic genetic features during ABC treatment. (A) Eleven patients had newly identified *TP53* mutations, and five patients lost *TP53* mutations. (B) Five patients had newly identified *NOTCH2* alterations, and one patient lost *NOTCH2* alterations. (C) Two patients had newly identified *GNAS* mutations, and three patients lost *GNAS* mutations. (D) Two patients had newly identified *TOP1* mutations, and one patient lost *TOP1* mutation. (E) Two patients lost *CTCF* mutations. (F) Two patients lost *NOTCH1* mutations.

During the serial monitoring, we also observed dynamic changes in those prognostic genetic alterations identified in our baseline cohort (Fig. 4). Of the 59 patients in the ctDNA monitoring cohort, 11 (18.64%) patients had newly identified *TP53* mutations, and 5 (8.47%) patients lost *TP53* mutations in their subsequent treatment. Although only two (3.39%) patients had altered *NOTCH2* at the initial ctDNA test after relapse and/or metastases, newly identified *NOTCH2* alterations were observed in five (8.47%) patients during follow‐up. *GNAS* and *TOP1* mutations showed similar evolutionary patterns to *NOTCH2*; however, the percentage of patients harboring *CTCF* and *NOTCH1* mutations were relatively low and their evolutionary patterns were stable.

We then investigated the relationship among ABC treatment plans, clinical response, and ctDNA dynamic changes in the ctDNA monitoring cohort (Table 3). The majority of patients received chemoradiotherapy or endocrine therapy at the beginning of their ABC treatment, followed by monoclonal antibody drugs, tyrosine kinase inhibitors, cell cycle inhibitors, or immunotherapy. The overall response rate decreased to < 10% after the fourth‐line ABC treatment. Patients conducted ctDNA tests more frequently when they received later‐line treatment, and the ctDNA positive rate kept increasing with the line of ABC treatment, from 59.46% to 85.29%. Additionally, patients were more likely to conduct ctDNA tests when they were treated with targeted drugs, TKIs, or cell cycle inhibitors. During the serial monitoring, *TP53* mutational status maintained negative among 21 (35.59%) patients and positive among 22 (37.29%) patients. *TP53* mutations were not detectable in at least one serial plasma samples among five (8.47%) patients who were positive for *TP53* mutations at the initial ctDNA test. A total of 11 (18.64%) patients were newly identified with *TP53* mutations, and 2 of them lost acquired *TP53* mutation later. These 11 patients were receiving chemotherapy or targeted therapy when new *TP53* mutations were detected, while none of them were receiving endocrine therapy. BC subtypes appeared to be not strongly associated with newly identified *TP53* mutations, and 3 of 11 patients showed subtype conversion (HR‐positive/HER2‐negative to TNBC; HER2‐enriched to HR‐positive/HER2‐negative; HR‐negative/HER2‐positive to TNBC; and then to HR‐negative/HER2‐positive). Intriguingly, 5 of the 11 (45.45%) patients had received or were receiving platinum chemotherapy when new *TP53* mutations were detected; however, platinum chemotherapy records were observed in 7 of 48 (14.58%) patients without newly identified *TP53* mutations.

**Table 3:** Advanced breast cancer treatment, clinical response, ctDNA, and *TP53* mutational status. Treatment – Endocrine therapy: Letrozole, Exemestane, Fulvestrant, etc. Platinum chemotherapy: Carboplatin, Cisplatin. Other chemoradiotherapy/surgery: Capecitabine, Docetaxel, Epirubicin, Doxorubicin, etc. HER2‐targeted therapy: Pertuzumab, Trastuzumab, Lapatinib, etc. Other targeted therapy: Everolimus, Apatinib, etc. Cell cycle inhibitor: Palbociclib, Ribociclib, etc. Immune therapy: Sintilimab, Camrelizumab, etc. CR, complete response; PR, partial response; SD, stable disease; PD, progressive disease.

Characteristics	ABC treatment lines					
	1st	2nd	3rd	4th	5th and later	Overall
Treatment, no. (%)						
Endocrine therapy	17 (28.81)	14 (25.45)	5 (10.20)	4 (9.30)	4 (3.70)	44 (14.01)
Platinum chemotherapy	5 (8.47)	2 (3.64)	3 (6.12)	1 (2.33)	9 (8.33)	20 (6.37)
Other chemoradiotherapy/surgery	19 (32.20)	22 (40.00)	22 (44.90)	19 (44.19)	33 (30.56)	115 (36.62)
HER2 targeted	12 (20.34)	11 (20.00)	7 (14.29)	13 (30.23)	32 (29.63)	75 (23.89)
Other targeted	2 (3.39)	5 (9.09)	8 (16.33)	4 (9.30)	16 (14.81)	35 (11.15)
Cell cycle inhibitor	4 (6.78)	0 (0.00)	4 (8.16)	1 (2.33)	8 (7.41)	17 (5.41)
Immune	0 (0.00)	0 (0.00)	0 (0.00)	1 (2.33)	6 (5.56)	7 (2.23)
Unknown	0 (0.00)	1 (1.82)	0 (0.00)	0 (0.00)	0 (0.00)	1 (0.32)
Total	59 (100.00)	55 (10.00)	49 (100.00)	43 (100.00)	108 (100.00)	314 (100.00)
Clinical response, no. (%)						
CR	1 (1.69)	1 (1.82)	0 (0.00)	0 (0.00)	0 (0.00)	2 (0.64)
PR	16 (27.12)	8 (14.55)	4 (8.16)	8 (18.60)	8 (7.41)	44 (14.01)
SD	16 (27.12)	15 (27.27)	16 (32.65)	12 (17.91)	34 (31.48)	93 (29.62)
PD	15 (25.42)	21 (38.18)	26 (53.06)	20 (46.51)	51 (47.22)	133 (42.36)
Undetected	1 (1.69)	1 (1.82)	1 (2.04)	2 (4.65)	10 (9.26)	15 (4.78)
Unknown	10 (16.95)	9 (16.36)	2 (4.08)	1 (2.33)	5 (4.63)	27 (8.60)
Total	59 (100.00)	55 (10.00)	49 (100.00)	43 (100.00)	108 (100.00)	314 (100.00)
ctDNA test frequency, no.	37	31	29	30	102	170
ctDNA test positive rate, %	59.46	61.29	79.31	80.00	85.29	76.39
Newly identified *TP53* mutation, no.	1	1	1	4	4	11

**Fig. 5:**
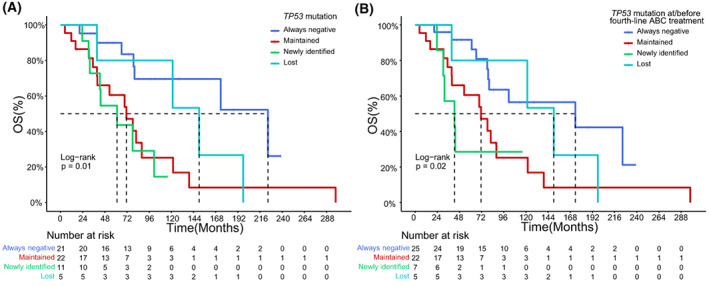
Overall survival, among patients with different evolutionary patterns of *TP53* mutations. Survival analysis using Kaplan–Meier was conducted. The broken line indicates the median OS of each subgroup. (A) Patients who maintained or had newly identified *TP53* mutations displayed poorer prognosis than patients without or losing *TP53* mutation (overall, *P* = 0.01; Always negative vs. Newly identified, *P* < 0.01). (B) When patients were classified according to their first four lines of ABC treatment, the association between the evolutionary pattern of *TP53* mutations and OS remained significant (overall, *P* = 0.02; Always negative vs. Newly identified, *P* < 0.01).

Patients in the ctDNA monitoring cohort were further classified into four subgroups according to the evolutionary pattern of *TP53* mutations during ABC treatment, including *TP53* mutation‐always negative group, *TP53* mutation‐maintained group, *TP53* mutation‐newly identified group, and *TP53* mutation‐lost group. The median OS of patients with maintained and newly identified *TP53* mutations were 71.4 and 61.2 months, respectively, shorter than that in patients who were always negative (224.5 months) or became negative with *TP53* mutation (150.0 months). The evolutionary pattern of *TP53* mutations was significantly associated with the OS of ABC patients (*P* = 0.01, Fig. 5A). Furthermore, compared to patients in the *TP53* mutation‐always negative group, patients with newly identified *TP53* mutations had significantly inferior OS (*P* < 0.01). To mitigate the confounding effect of time at which *TP53* mutation was acquired, we also investigated the dynamic change of *TP53* mutational status at/before the fourth‐line treatment. Similar to the results derived from the whole ABC treatment history, the relationship between *TP53* mutation evolutionary pattern and OS remained statistically significant when we restricted the time within four lines of treatment (*P* = 0.02, Fig. 5B), suggesting that newly identified *TP53* mutation was a negative biomarker for ABC patients' OS. However, neither significantly higher VAFs of all mutant genes (Fig. S1A) nor VAFs of mutant genes other than *TP53* (Fig. S1B) were observed in the *TP53* mutation‐newly identified group, in comparison with *TP53* mutation‐always negative group or *TP53* mutation‐lost group. For the maximum VAF during ctDNA monitoring, similar results were obtained (Fig. S1C,D), suggesting that the disease burden might not be different across these three subgroups. Of note, compared to mutation‐always negative group, the mutation‐maintained group had significantly higher maximum VAFs of all mutant genes (*P* < 0.01) and VAFs of mutant genes other than TP53 (*P* < 0.01). Furthermore, the expansion of the *TP53* clone was observed in three patients with newly identified *TP53* mutations (Fig. S2A). In another six patients in the *TP53* mutation‐newly identified group, the dynamic patterns of *TP53* mutation VAF and maximum VAF were highly correlated, even though *TP53* clones might not be dominant clones in these patients (Fig. S2B). In the remaining two patients, new *TP53* mutations were also identified when maximum VAF declined (Fig. S2C).

## 4 Discussion

Our data revealed the prognostic value of a group of baseline genetic features and *TP53* mutation evolutionary pattern in ABC. Patients carrying genetic alterations of *TP53*, *CTCF*, *GNAS*, and *NOTCH1* in baseline tumor tissues were associated with poor prognosis. Our findings on mutant *TP53* gene in the baseline cohort were consistent with previous studies [38, 39]. Few studies identified that *CTCF* mutation was a prognostic factor for BC, even though the ability of *CTCF* of binding to the promoter/insulator sites of cell proliferation‐related genes could be influenced by *CTCF* gene mutation, and one isoform of *CTCF* protein (130 kDa) was identified as a BC biomarker [40]. Similarly, abnormal *GNAS* expression level was associated with the promotion of BC cell proliferation, distal metastasis, and poor OS, while genomic *GNAS* mutation was not clearly identified as a prognostic factor in BC [41]. In the baseline cohort, we could not separately estimate the impact of *CTCF* and *GNAS* mutation on DFS, and other mutated genes related to BC, such as *PIK3CA*, *PTEN*, *BRCA2*, were also identified in these three patients who carried both *CTCF* and *GNAS* mutations. Thus, we supposed that the negative influence might be partially attributed to mutant *CTCF* and *GNAS* genes. Moreover, our finding on the prognostic potential of *NOTCH* gene was also consistent with previous research. One meta‐analysis involving 3867 patients showed that BC patients with high‐level *NOTCH1* expression displayed significantly inferior relapse‐free survival and OS [42]. Worse PFS was observed among Hispanic Latina female TNBC patients harboring *NOTCH* pathway gene mutations [43].

*TP53* alternations were more frequently detected among HER2‐enriched and TNBC patients in our baseline cohort. The enrichment of *TP53* mutation was also observed in one European cohort of 1794 BC patients and one Chinese cohort of 411 BC patients [43, 44]. *ZNF703*, *CTCF*, and *GNAS* alterations were exclusively observed among HR‐positive/HER2‐negative patients, which was consistent with what was observed in a study on 11 616 breast tumors. *ZNF703* and *GNAS* alterations were more commonly detected among ER‐positive/HER2‐negative tumors than HR‐negative/HER2‐negative or HER2‐positive tumors [45]. Furthermore, this study indicated that *CTCF* mutations were enriched in tumor samples of metastatic BC compared to local BC (2% vs. 0.9%, *P* < 0.01), and it was supposed that *CTCF* mutation might be a metastatic driver [45]. Compared to local BC samples in the previous study, in our study, we observed the proportion of tissue samples carrying *CTCF* mutations was higher in the baseline cohort, in which all patients ultimately experienced metastasis (11.43% [4/35] vs. 0.86% [39/4512], *P* < 0.01). Thus, we supposed that BC tumor with *CTCF* mutations were more likely to develop metastasis, even though the number of patients in the baseline cohort was limited.

The evolutionary pattern of *TP53* mutation was the most interesting finding in the ctDNA monitoring cohort. We explored the association of newly identified *TP53* mutations during ABC treatment with treatment history, BC subtypes, and subtype conversion. Although it had been reported that acquired *TP53* mutations could be induced by platinum chemotherapy in ovarian cancer as a result of drug resistance [46, 47], it was hard for us to confirm the causality in ABC. Patients in this real‐world study had extremely complicated treatment history and their ctDNA test schedules rarely matched their ABC treatment schedules, which means it was possible for other previous ABC treatments to induce newly identified *TP53* mutations.

Our data also revealed the prognostic value of the newly identified *TP53* mutation, and emphasized the importance of monitoring *TP53* mutation by liquid biopsy profiling during ABC treatment. Presentence of newly identified *TP53* mutations were detected in 11 of 59 patients, while these patients did not show significantly higher VAF/maximum VAF than patients in the *TP53* mutation always negative or *TP53* mutation lost group, suggesting that *TP53* clone might be a potential indicator of poorer OS, independent of VAF/maximum VAF. Similar to a previous treatment‐emergent alteration study in which the VAF of secondary *RAS* was compared to the VAF of *KRAS* ^*G12C*^ mutation [48], we further compared the dynamics of *TP53* mutation VAF and maximum VAF in patients with newly identified *TP53* mutations. We supposed that the rising *TP53* VAF mainly resulted from *TP53* clone expansion, while the increasing disease burden could not be completely ignored. However, it remained an interesting issue whether prognosis of patients with newly identified *TP53* mutations during ABC treatment could be further stratified depending on these two potential mechanisms. This retrospective real‐world study had serval limitations. For instance, well‐planned liquid biopsy schedules were rare among our patients, resulting in missing records on *TP53* mutational status and the misclassification of patients, especially those with long follow‐up. Additionally, we were also unable to explore the potential confounding effect of BC subtypes due to the limited sample size of patients with newly identified *TP53* mutation. The dynamic clinical characteristics during ABC treatment (e.g., BC subtype conversion, complicated treatment plans, and metastasis development) also made our results to be interpreted more carefully [49, 50]. Alternatively, time‐dependent Cox regression analysis could be used to address this limitation [51], whereas a larger ctDNA monitoring cohort with more ctDNA analysis data would be needed.

## 5 Conclusion

In summary, we conducted a retrospective study on ABC patients experiencing relapse and/or metastases. Four baseline tumor genetic features, including *TP53*, *CTCF*, *GNAS*, and *NOTCH1*, were identified as potential prognostic factors of ABC. The newly identified *TP53* mutation in liquid biopsies during ABC treatment was associated with poor prognosis, and the evolutionary pattern of *TP53* mutation could potentially serve as a prognostic factor for recurrent BC patients.

## Conflict of interest

XZ, JL, YX, RY, and QO are employees of Nanjing Geneseeq Technology Inc., China. The remaining authors have nothing to disclose.

## Author contributions

ZZ and ML designed the study and supervised the work. LZ, SS, XZ, and JL conceived and designed the study. LZ collected and collated the clinical data. LZ, SS, LX, CS, NL, JY, SZ, PY, FF, JX, and XJ collected clinical samples (tumor tissue and peripheral blood) for NGS analyses. LZ, SS, XZ, JL, YX, RY, and QO analyzed and interpret the data. LZ, XZ, JL, YX, RY, and QO wrote the manuscript. ZZ, ML, and YX reviewed and revised the manuscript. All authors have read and approved the final manuscript.

## Supporting information


**Fig. S1.** Variant allele frequencies (VAF) of patients in the circulating tumor DNA (ctDNA) monitoring cohort.

**Fig. S2.** The fluctuation of *TP53* mutation and maximum variant allele frequency in 11 patients with newly identified *TP53* mutations.

**Table S1.** Baseline *TP53* mutation in relation to hormone receptor status.

**Table S2.** Genetic alterations identified in baseline samples in this study.

**Table S3.** Genetic alterations identified in serial blood samples in this study.

Data S1

## Data Availability

The data that support the findings of this study are available from the corresponding author man_li@dmu.edu.cn upon reasonable request.
