# Supplementary figures and images for: Prognostic value of baseline genetic features and newly identified TP53 mutations in advanced breast cancer
Source: Mol Oncol. 2022 Aug 15;16(20):3689–702. doi: 10.1002/1878-0261.13297 (PMC9580879; doi:10.1002/1878-0261.13297)

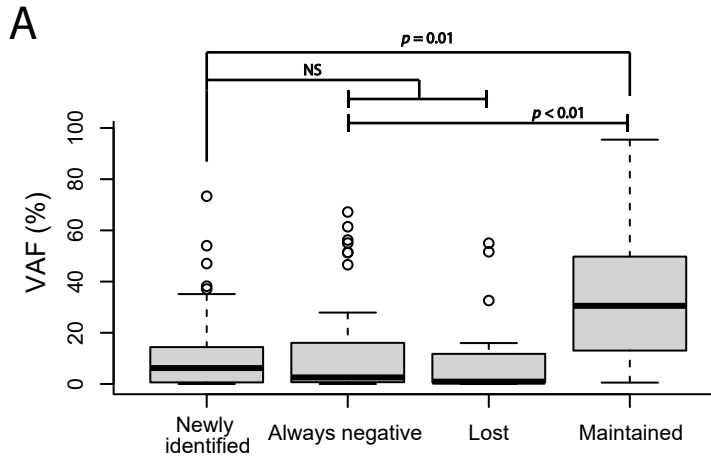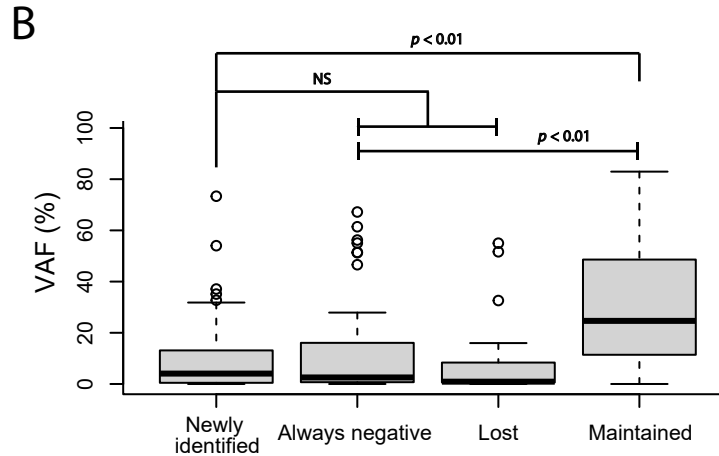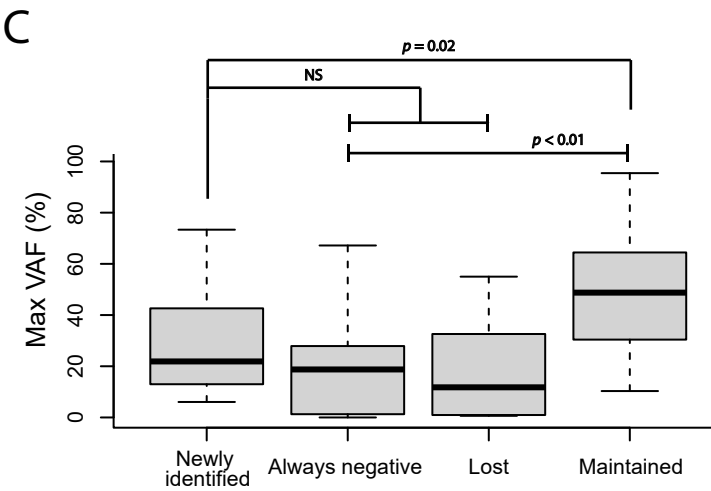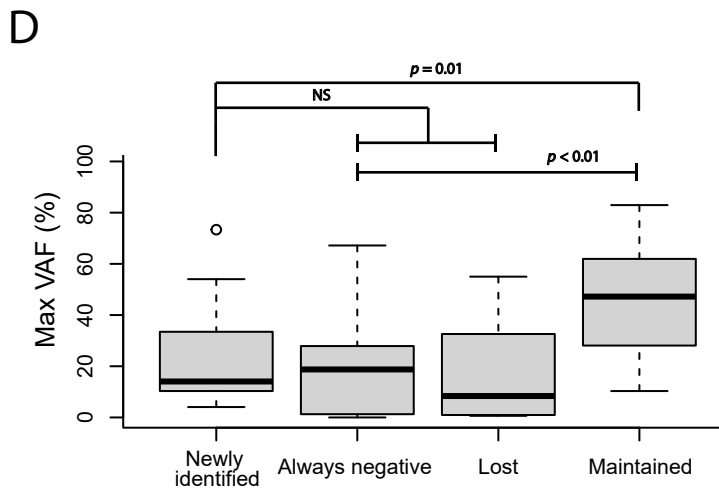

Supplement: Supplementary file 1 — Fig. S1. Variant allele frequencies (VAF) of patients in the circulating tumor DNA (ctDNA) monitoring cohort. [file MOL2-16-3689-s002.pdf]

A

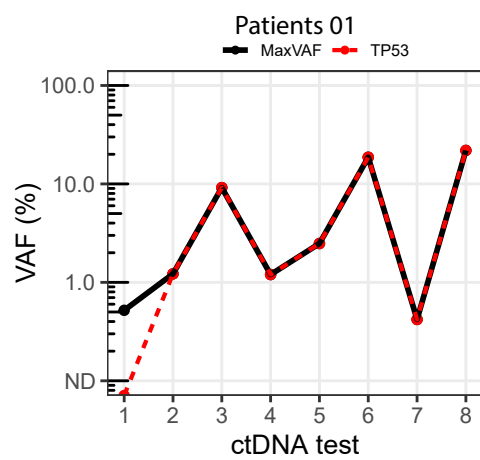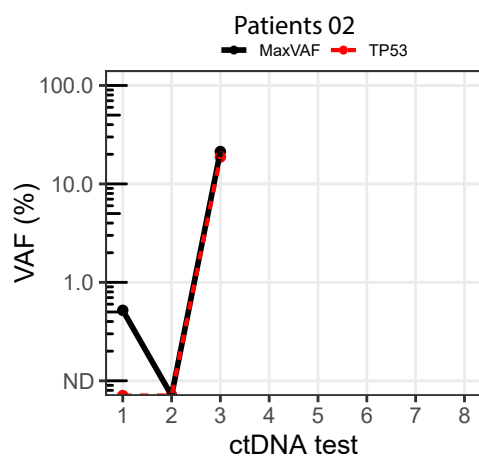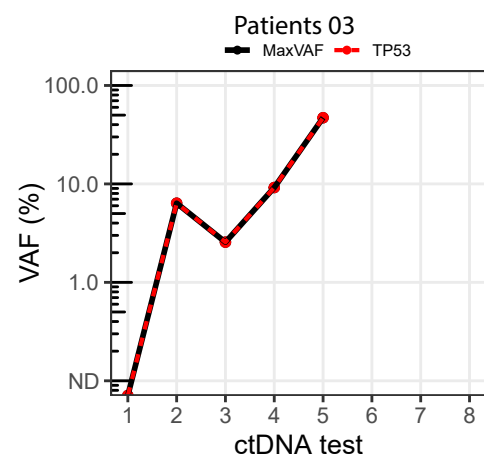

B

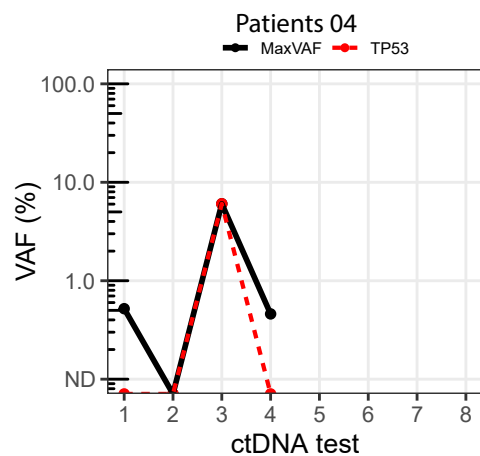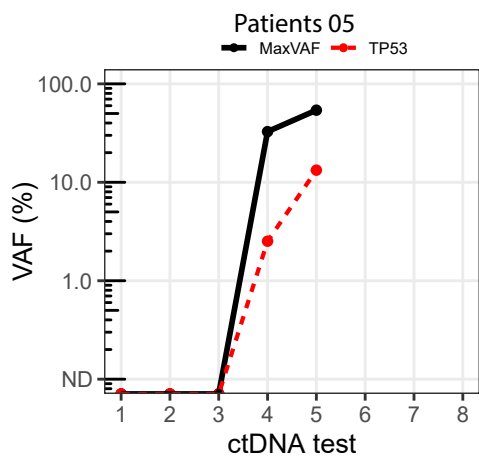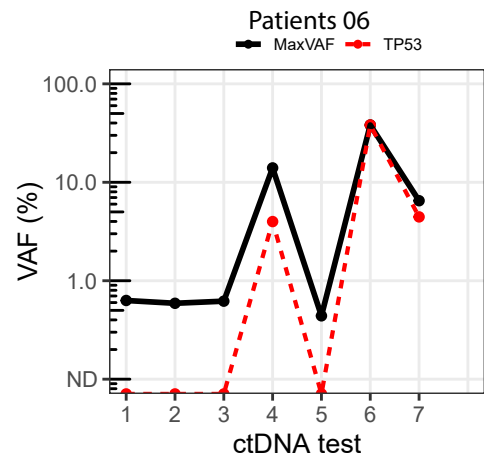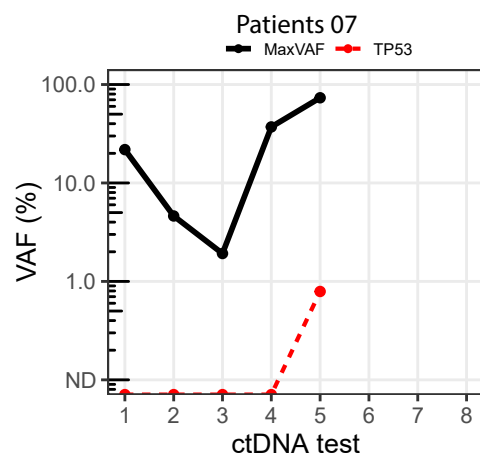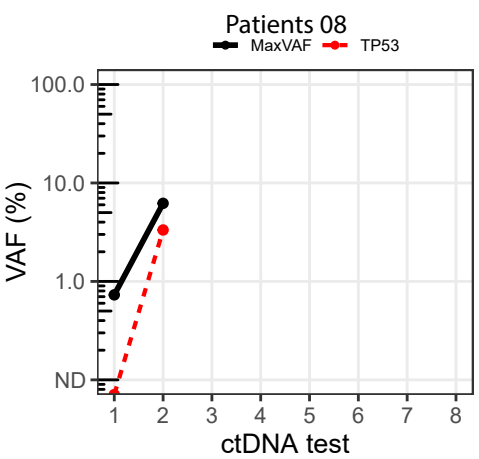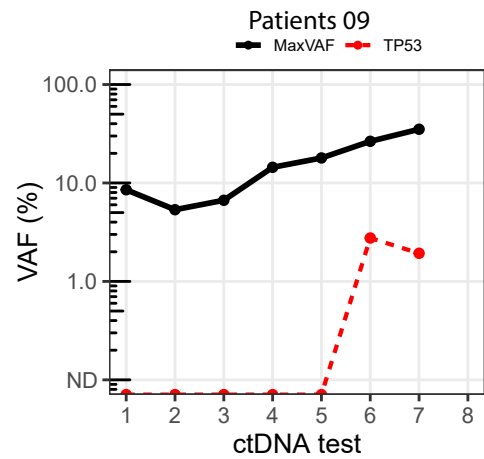

C

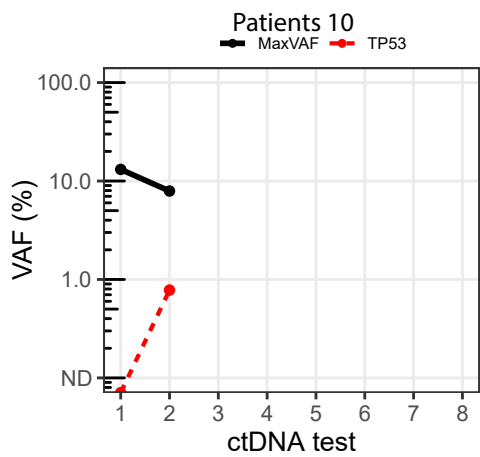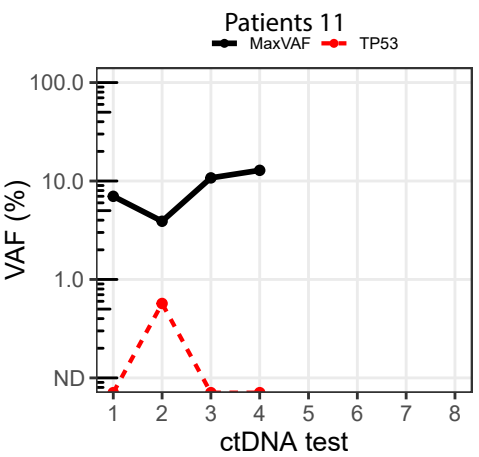

Supplement: Supplementary file 2 — Fig. S2. The fluctuation of TP53 mutation and maximum variant allele frequency in 11 patients with newly identified TP53 mutations. [file MOL2-16-3689-s004.pdf]
