# Supplementary material for: Prognostic value of baseline genetic features and newly identified TP53 mutations in advanced breast cancer
Source: Mol Oncol. 2022 Aug 15;16(20):3689–702. doi: 10.1002/1878-0261.13297 (PMC9580879; doi:10.1002/1878-0261.13297)
Supplement: Supplementary file 6 — Data S1 [file MOL2-16-3689-s006.docx]

**Supplementary Figure S1.** Variant allele frequencies (VAF) of patients in the circulating tumor DNA (ctDNA) monitoring cohort. (A) VAF of all plasma samples tested during the ctDNA monitoring. Patients in the newly identified subgroup had similar VAF to patients in the always negative subgroup or the lost group, while the maintained subgroup had higher VAF than the always negative subgroup. (B) Similar results were obtained when we focused on the VAF of somatic mutations other than *TP53* gene. (C) Maximum VAF of each patient during the ctDNA monitoring. Patients in the newly identified subgroup had similar maximum VAF to patients in the always negative subgroup or the lost group, while the maintained subgroup had a higher maximum VAF than the always negative subgroup. (D) Similar results were obtained when we focused on the maximum VAF of somatic mutations other than *TP53* gene.

**Supplementary Figure S2.** The fluctuation of *TP53* mutation and maximum variant allele frequency in 11 patients with newly identified *TP53* mutations. (A) The *TP53* clone was identified as the dominant clone in three patients. (B) The rise of *TP53* mutation could potentially indicate worsening disease of advanced breast cancer in six patients. (C) *TP53* clone expansion might be independent of the overall disease burden in two patients.
